# Regulatory mechanisms and research progress of astrocytes in the neuro-endocrine-immune network

**DOI:** 10.3389/fnhum.2026.1865645

**Published:** 2026-09-02

**Authors:** Xinhui Cheng, Yang Guo, Yupei Cheng, Ning Yu, Yiping Sun, Shenjun Wang, Yi Guo

**Affiliations:** 1 School of Acupuncture & Moxibustion and Tuina, Tianjin University of Traditional Chinese Medicine, Tianjin, China; 2 Research Center of Experimental Acupuncture Science, Tianjin University of Traditional Chinese Medicine, Tianjin, China; 3 First Teaching Hospital of Tianjin University of Traditional Chinese Medicine, Tianjin, China; 4 National Clinical Research Center for Chinese Medicine, Tianjin, China; 5 Department of Tranditional Chinese Medicine, Qinghai Unversity Medical College, Xining, China

**Keywords:** astrocytes, calcium signaling, extracellular vesicles, metabolic homeostasis, neuro-endocrine-immune network, neurovascular coupling, reactive astrocytes

## Abstract

The neuro-endocrine-immune (NEI) network is a key regulatory network that maintains organismal homeostasis and participates in stress responses. Its function depends on the dynamic coupling among the nervous, endocrine, and immune systems. Astrocytes, as abundant and functionally diverse glial cells in the central nervous system, are increasingly recognized as important participants in the NEI network. Growing evidence indicates that astrocytes do not merely provide structural support in the traditional sense, but also exhibit marked regional heterogeneity and functional diversity. Through broad sensing of neural, endocrine, and immune signals, gliotransmitter release, metabolic regulation, extracellular vesicle-mediated communication, and interactions with neurons, glial cells, the glymphatic system, and endocrine axes, astrocytes actively participate in the integration of neural activity, the regulation of endocrine homeostasis, and immune responses. Under physiological conditions, astrocytes contribute to the dynamic balance of the NEI network by integrating multisource signals derived from neurons, endocrine factors, and immune molecules. Under pathological conditions, such as inflammation, ischemia, and neurodegenerative diseases, the phenotype and function of astrocytes undergo substantial remodeling, thereby contributing to NEI network dysregulation and disease progression. In this structured narrative review, literature published between 2016 and 2026 was retrieved from the PubMed and Web of Science databases using “astrocytes,” “nervous,” “endocrine,” and “immune” as search terms. It summarizes the structural and functional basis of astrocytes and the mechanisms by which they regulate the NEI network, with the aim of systematically elucidating the physiological and pathological significance of astrocytes in NEI network integration and providing a theoretical basis for mechanistic studies and targeted intervention strategies for related diseases.

## 1 Introduction

The neuro-endocrine-immune (NEI) network constitutes a fundamental basis for adaptive regulation in the body. At its core, this network maintains internal homeostasis through information exchange among the nervous, endocrine, and immune systems (Xie et al., 2025). In recent years, the role of astrocytes within this network has attracted increasing attention.

Traditionally, astrocytes have been regarded as glial cells that maintain microenvironmental homeostasis in the central nervous system, with major functions including regulation of ion concentrations, modulation of synaptic transmission, and participation in the formation and functional maintenance of the blood–brain barrier (Djukic et al., 2007; Panatier et al., 2011; Alvarez et al., 2011). However, this view has been continuously revised by accumulating evidence in recent years. At the neuroendocrine level, astrocytes can not only sense hormonal changes but also regulate the activity of neurosecretory neurons, thereby influencing neurosecretion. Studies have shown that, in key neuroendocrine regions such as the hypothalamus, astrocytes participate in the regulation of hypothalamic neuronal activity by sensing insulin signaling, thereby modulating systemic energy homeostasis (García-Cáceres et al., 2016). In addition, hypothalamic astrocytes can regulate neuronal activity through adenosine A1 receptor signaling and consequently influence feeding behavior (Yang et al., 2015). These findings suggest that astrocytes possess functions related to endocrine signal sensing, regulation of neuronal activity, and modulation of neurosecretory output in neuroendocrine regulation, making them important cellular mediators linking neuronal activity with peripheral endocrine effects.

At the neuroimmune level, astrocytes have been shown to actively respond to inflammatory responses in the central nervous system. One study demonstrated that interleukin-1α (IL-1α), tumor necrosis factor (TNF), and complement component 1q (C1q) released by activated microglia can induce neurotoxic reactive astrocytes, indicating that astrocytes can directly respond to inflammatory mediators and undergo functional remodeling (Liddelow and Barres, 2017). Further research revealed that microglia can regulate astrocyte reactivity in response to gut microbiota-derived metabolites, thereby influencing central nervous system inflammation, suggesting that peripheral metabolic and immune signals may also shape the inflammatory milieu in the brain through glial cells (Rothhammer et al., 2018). From the perspective of transcriptional regulation, another study found that MAF bZIP transcription factor G (MAFG) promotes the transition of astrocytes toward a pro-inflammatory phenotype, thereby exacerbating inflammatory responses in the central nervous system (Wheeler et al., 2020). In addition to cytokines, damage-associated nucleotide signaling is also an important mediator of immune communication in the brain. Adenosine triphosphate (ATP) signaling has been shown to rapidly mediate microglial chemotaxis toward sites of focal brain injury, contributing to the microglial immune response to local damage (Davalos et al., 2005). Moreover, astrocyte-derived ATP signals generated after injury can encode local injury information and be detected and responded to by microglia (Chen et al., 2024). Taken together, astrocytes not only participate in the regulation of neuronal activity and metabolic homeostasis but also respond to inflammatory cytokines, peripheral metabolic signals, and damage-associated nucleotide signals, thereby serving as a bridge among neural activity, endocrine regulation, and immune responses. Therefore, we propose that the conceptual understanding of astrocyte function has gradually evolved from that of passive supportive cells in the traditional sense to that of an “integrative node” within the NEI network.

These findings suggest that, compared with neurons, which primarily mediate information transmission, and microglia, which mainly perform immune surveillance, astrocytes may be better suited to serve as key integrative nodes within the NEI network. This capacity is attributable to their multimodal signal-sensing abilities, their spatial advantage in simultaneously interfacing with neurons and blood vessels, and their regulatory effects on neuronal activity and the surrounding microenvironment. Therefore, systematically elucidating the mechanisms by which astrocytes regulate the NEI network will not only advance our understanding of the biological principles underlying astrocyte-mediated maintenance of brain homeostasis, but also provide a theoretical basis for developing new therapeutic strategies that target astrocytes as potential intervention points.

## 2 Literature search and organization

**Figure 1:**
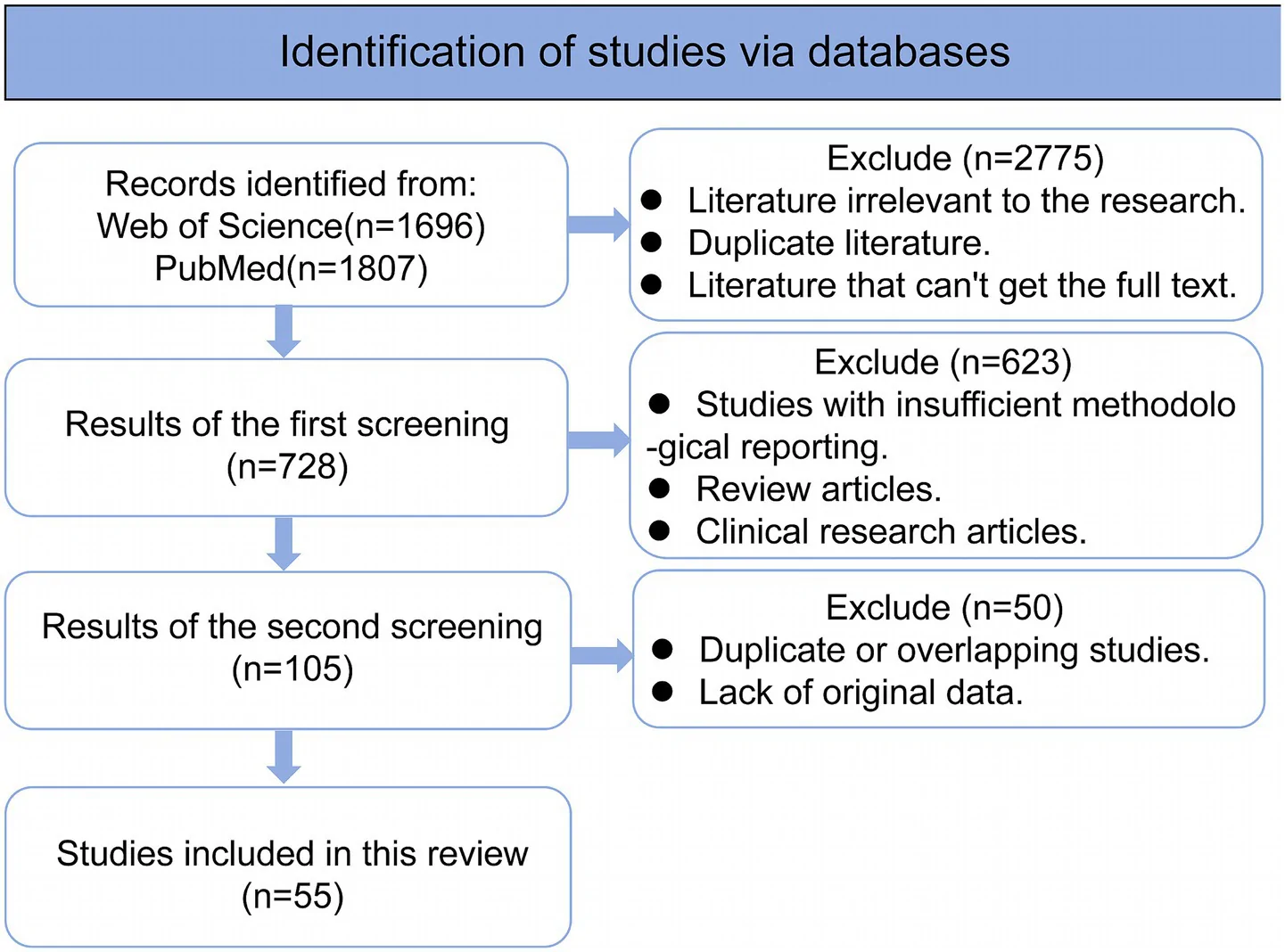
Search process.

Using “astrocytes,” “neural,” “endocrine,” and “immune” as search terms, a literature search was conducted in the PubMed and Web of Science databases for studies published between 2016 and 2026. The initial search identified 1,807 records in PubMed and 1,696 records in Web of Science. Ultimately, 55 articles were included for analysis. This review was designed as a structured narrative review rather than a systematic review or scoping review. The primary search was limited to articles published between 2016 and 2026. Earlier foundational studies were selectively cited when they reported seminal discoveries, established key concepts, or introduced methodological advances essential for understanding astrocyte biology and neuro-endocrine-immune regulation. These earlier publications were identified through reference tracing from key articles and expert knowledge of the field and were used mainly to provide background and conceptual context rather than as part of the principal synthesis set. Review articles and studies examining human brain tissues, disease-associated samples, or human-derived astrocyte models were excluded from the principal mechanistic synthesis. Nevertheless, selected review articles were used to provide background information, summarize established concepts, and identify additional relevant primary studies through reference tracing. A limited number of studies examining human brain tissues, disease-associated samples, or human-derived astrocyte models were cited when relevant to disease context or translational implications; however, they were not considered primary mechanistic evidence in the core synthesis. The search process is described in Figure 1.

## 3 Regional heterogeneity and functional diversity of astrocytes

Astrocytes are abundant, widely distributed, and functionally diverse glial cells in the central nervous system. With the rapid development of single-cell transcriptomics, spatial omics, and cell type–specific protein markers, the heterogeneity and functional specialization of astrocyte subpopulations have been characterized in a more systematic and refined manner at the transcriptional, protein, and spatial distribution levels (Hennes et al., 2025; Prabhakar et al., 2023). Traditionally, astrocytes were generally regarded as simple supportive cells whose primary functions were to maintain ionic homeostasis, clear neurotransmitters, and provide metabolic support. However, accumulating evidence indicates that astrocytes are not uniformly distributed throughout the central nervous system. Instead, they exhibit pronounced regional specificity, with their morphological features, molecular expression profiles, and physiological functions depending strongly on brain region, neural circuitry, and developmental stage (Chai et al., 2017; Schober et al., 2022; Endo et al., 2022).

From the perspectives of molecular phenotype and cellular architecture, astrocytes in different regions differ markedly in transcriptomic composition, protein expression, morphological characteristics, and functional properties, suggesting substantial regional specificity and functional diversity (Hennes et al., 2025; Chai et al., 2017; Kwon et al., 2025). For example, astrocytes in the hippocampal dentate gyrus display distinct molecular markers, morphological structures, and physiological responses that distinguish them from astrocytes in adjacent regions (Karpf et al., 2022). Astrocytes in white matter and the optic nerve head form subpopulations with specific structural and functional properties, primarily contributing to the regulation of local mechanical stress, axonal support, and maintenance of white matter homeostasis (Mazumder et al., 2022; Bocchi et al., 2025; Bugiani et al., 2022). These findings indicate that the regional specificity of astrocytes not only reflects differences in anatomical distribution but also provides an important structural basis for maintaining the functions of local neural networks. Accordingly, regional astrocyte heterogeneity has become an important entry point for understanding functional specialization across brain regions and the regulation of local homeostasis. Importantly, regional astrocyte heterogeneity is not a static or intrinsic property, but rather a dynamic process that emerges through the combined effects of developmental programming, local signaling, and neuronal activity. Studies have shown that primary cilium–mediated signal transduction in astrocytes plays a key role in their developmental maturation and region-specific functions, suggesting that intrinsic cellular structural factors have an important influence on astrocyte functional specialization (Wang et al., 2024). At the same time, neuronal activity, vascular signals, the synaptic microenvironment, interactions among glial cells, and disease-related stimuli can continuously reshape the functional and morphological characteristics of astrocytes (Endo et al., 2022; Bartels et al., 2024; Ben Haim and Rowitch, 2016; Torres-Ceja and Olsen, 2022). Therefore, astrocyte heterogeneity should be understood as a dynamic functional state rather than merely a morphological variation.

The regional heterogeneity of astrocytes endows them with diverse functions. In higher cognitive regions such as the hippocampus, astrocytes can regulate neuronal excitability and synaptic homeostasis, thereby contributing to learning, memory, and information processing (Chai et al., 2017; Karpf et al., 2022; Ben Haim and Rowitch, 2016). Astrocytes in perivascular regions provide structural support and participate in metabolic coupling, neurovascular coupling, and the regulation of cerebrospinal fluid circulation, all of which are essential for neural tissue homeostasis and substance exchange (Cohen-Salmon et al., 2025; Li et al., 2025; Gao et al., 2023). Astrocytes in the substantia gelatinosa of the spinal dorsal horn are involved in pain sensitization and central nervous system inflammation (Ficker et al., 2014). In neuroendocrine centers such as the hypothalamus, astrocytes can sense hormonal signals and participate in glucose metabolism and energy balance by influencing neuronal excitability and the local microenvironment, suggesting a critical role for astrocytes in neuroendocrine regulation (García-Cáceres et al., 2016; Herrera Moro Chao et al., 2022). Thus, astrocytes provide an important structural basis and functional support for the regulation of the NEI network (Region-specific functions of astrocytes in different brain areas in Table 1).

**Table 1:** Region-specific functions of astrocytes in different brain areas.

Anatomical region	Region-specific functions	References
Hippocampus	They clear neurotransmitters, buffer extracellular ions, regulate neuronal excitability, maintain local metabolic and ionic homeostasis, and participate in synaptic plasticity and hippocampal information processing.	Chai et al. (2017), Karpf et al. (2022), Ben Haim and Rowitch (2016)
Hypothalamus	They sense hormonal signals and participate in glucose metabolism and energy homeostasis by modulating neuronal excitability and the local microenvironment, thereby playing a key role in neuroendocrine regulation.	García-Cáceres et al. (2016), Herrera Moro Chao et al. (2022)
White matter	They maintain the ion and water homeostasis required for axonal conduction, provide metabolic support, regulate oligodendrocyte lineage and myelin homeostasis, and mediate specific responses in white matter pathologies such as demyelination, ischemia, and inflammation.	Bocchi et al. (2025), Bugiani et al. (2022)
Optic nerve head	They contribute to local structural support, maintenance of axonal microenvironmental homeostasis, and responses to mechanical stress, and may play an important role in optic nerve injury–related diseases such as glaucoma.	Mazumder et al. (2022)
Perivascular area	They provide structural support and are involved in metabolic coupling, neurovascular coupling, and the regulation of cerebrospinal fluid circulation.	Cohen-Salmon et al. (2025), Li et al. (2025), Gao et al. (2023)
Substantia gelatinosa	They are involved in pain sensitization and central neuroinflammation.	Ficker et al. (2014)

## 4 Mechanisms underlying the role of astrocytes as hubs in the NEI network

### 4.1 Sensing of neural, endocrine, and immune signals by astrocytes

A The ability of astrocytes to participate in the regulation of the NEI network depends on their diverse capacity for signal sensing. Current evidence indicates that astrocytes express a wide range of membrane receptors, ion channels, and intracellular signaling molecules, enabling them to detect extracellular signals such as neurotransmitters and to regulate neuronal activity and the local microenvironment through Ca^2+^ dynamics and related signaling pathways (Guttenplan et al., 2025; Li et al., 2020; Chen et al., 2025; Zhang et al., 2023). At the level of neural signaling, astrocytes can sense synaptic activity through γ-aminobutyric acid (GABA)-mediated signaling pathways and G protein-coupled receptor (GPCR) systems, and other neurotransmitter-related receptors. They translate neuronal firing and local network states into intracellular Ca^2+^ dynamics, metabolic responses, and gene-expression programs, thereby contributing to the regulation of synaptic transmission, neuronal excitability, and local microenvironmental homeostasis (Guttenplan et al., 2025; Zhang et al., 2023; Mederos et al., 2021; Liu et al., 2022; Ameroso and Rios, 2024). In endocrine regulation, astrocytes also occupy a critical sensing position. In particular, within the hypothalamus, a key neuroendocrine hub, astrocytes participate in the regulation of energy metabolism, glucose homeostasis, and circadian rhythm-related processes (Herrera Moro Chao et al., 2022; MacDonald et al., 2019; Schouten et al., 2025; Tran et al., 2023). Studies have shown that hypothalamic astrocytes can detect changes in insulin, glucocorticoids, estrogen, and prolactin, and contribute to the maintenance of glucose homeostasis and energy balance by modulating the activity of neighboring neurons (García-Cáceres et al., 2016; Lu et al., 2020; Castillo et al., 2024; Lai et al., 2024; Maatouk et al., 2019).

With respect to immune signal recognition, astrocytes can detect inflammatory mediators such as tumor necrosis factor alpha(TNF-α), IL-1α, and C-X-C motif chemokine ligand 13(CXCL13) and rapidly undergo reactive functional remodeling. In addition, inflammatory stimulation can induce astrocytes to produce immune effector molecules, including prostaglandin E2(PGE2), interleukin-6(IL-6), and interleukin-15(IL-15), thereby contributing to the amplification and maintenance of inflammatory responses and indicating their high sensitivity within the inflammatory microenvironment (Nakajima et al., 2022; Li et al., 2017; Codeluppi et al., 2014; Shaw et al., 2026; Jiang et al., 2016). Astrocytes not only sense immune signals but also, to some extent, encode inflammatory events. Previous studies have shown that, after brain injury, the spatiotemporal dynamics of astrocytic ATP can serve as a form of injury-information encoding, which is recognized by microglia and triggers subsequent immune responses (Chen et al., 2024). This finding suggests that astrocytes do not merely respond passively to the local inflammatory microenvironment; rather, by releasing nucleotide signaling molecules such as ATP, they can transmit injury-related information to microglia and participate in the regulation of downstream immune responses.

In summary, astrocytes are not passive effector cells within a single signaling pathway; rather, they are capable of sensing neural, endocrine, and immune signals, thereby serving as hubs in the maintenance of NEI network homeostasis (Astrocytes sense neural, endocrine, and immune-related signals in Table 2).

**Table 2:** Astrocytes sense neural, endocrine, and immune-related signals.

Signals sensed by astrocytes	Experimental model	References
Insulin	Mice with insulin receptors specifically knocked out in astrocytes	García-Cáceres et al. (2016)
GPCR-mediated signaling	Mice cortical *in vivo* model with astrocyte-specific GPCR manipulation	Guttenplan et al. (2025)
GABA signaling	Mice prefrontal cortex model	Mederos et al. (2021)
Neuron-derived estrogen signaling	Mice with MCAO-induced focal cerebral ischemia	Lu et al. (2020)
Prolactin	Rats with MCAO-induced focal ischemic stroke	Castillo et al. (2024)
Glucocorticoid	MPTP-treated mice with astrocyte-specific glucocorticoid receptor manipulation	Maatouk et al. (2019)
TNF-α, IL-1α	Primary mouse astrocytes stimulated with TNF-α and IL-1α *in vitro*	Nakajima et al. (2022)
IL-15	Mice subjected to transient middle cerebral artery occlusion with astrocyte-specific IL-15 manipulation	Li et al. (2017)
CXCL13	Mice with neuropathic pain	Jiang et al. (2016)

### 4.2 Astrocytic information output: neurotransmitter release, vesicular secretion, and metabolic regulation

After integrating neural, endocrine, and immune signals, astrocytes do not merely remain at the level of signal sensing; rather, they further actively regulate the NEI network through diverse modes of information output. Numerous studies have shown that astrocytic outputs are highly heterogeneous, including not only the release of classical gliotransmitters but also extracellular vesicle-mediated intercellular communication, as well as processes such as metabolic substrate transport and energy coupling (Prada et al., 2011; Datta Chaudhuri et al., 2020; Bonvento and Bolaños, 2021).

At the level of neuromodulation, astrocytes can regulate synaptic transmission, neuronal excitability, and synaptic plasticity by releasing bioactive molecules such as glutamate, ATP, and GABA (Liu et al., 2022; Cuellar-Santoyo et al., 2022; Chen et al., 2022; Li et al., 2025; Illes et al., 2019). For example, astrocyte-derived glutamate can participate in the modulation of excitatory synaptic activity, whereas GABA can mediate inhibitory regulation, thereby influencing the balance of neural circuits (Liu et al., 2022; Cuellar-Santoyo et al., 2022). ATP released by astrocytes is also an important signaling molecule in the central nervous system and can modulate neuronal excitability and neural activity (Illes et al., 2019). In addition to the release of classical transmitters, connexin 43 (Cx43) hemichannels provide an important structural basis for astrocytic signal output. Studies have shown that various inflammatory stimuli can regulate Cx43 hemichannel activity, thereby affecting astrocytic Ca^2+^ dynamics and the release of related signaling molecules (Panattoni et al., 2021). Furthermore, extracellular vesicles (EVs) have been recognized as one of the key pathways through which astrocytes mediate long-distance communication and intercellular regulation. Growing evidence indicates that astrocyte-derived EVs can carry proteins, lipids, and inflammation-related molecules, thereby transmitting biological information among neurons, microglia, and even vascular-associated cells (Datta Chaudhuri et al., 2020; Rong et al., 2021; Crivelli et al., 2023). Importantly, the cargo transported by EVs is not static; instead, it can be remodeled according to the type of stimulus, the degree of inflammation, and the pathological context. Consequently, the effects of EVs on target cells may be either protective or toxic. For instance, vesicles released by activated astrocytes can regulate neuronal uptake, differentiation, and firing, whereas under pathological conditions, certain vesicles may carry proinflammatory or mitochondria-toxic components, thereby exacerbating neuroinflammation and neuronal injury (Crivelli et al., 2023; You et al., 2020).

In addition, astrocytes participate in information output within the NEI network through metabolic regulation. Through glucose uptake, lactate shuttling, lipid metabolism, and the glutamate–glutamine cycle, astrocytes provide energetic substrates for neuronal activity and maintain ionic homeostasis (Herrera Moro Chao et al., 2022; Zhang et al., 2023; Mi et al., 2023). In the hypothalamus and related metabolic circuits, the metabolic state of astrocytes can directly influence appetite regulation, energy expenditure, and glucose homeostasis; thus, their metabolic output essentially represents a form of neuroendocrine regulation (Herrera Moro Chao et al., 2022; Tran et al., 2023). Meanwhile, impairment of mitochondrial fatty acid degradation in astrocytes not only compromises energy supply but may also trigger metabolic-immune reprogramming, thereby promoting neuroinflammation and neurodegenerative changes (Mi et al., 2023). Therefore, astrocytic information output is not limited to the secretion of signaling molecules; the metabolic state of astrocytes itself also represents an important mechanism for regulating the neural and immune microenvironment. Because metabolic processes are tightly coupled with immune regulation, astrocytes can integrate neural activity, hormonal changes, and inflammatory stimuli on the basis of metabolic reprogramming.

Furthermore, studies suggest that mitochondrial transfer may represent another important form of astrocytic information output. In ischemic brain disorders, astrocytes can release functional mitochondria to neighboring neurons, helping to restore impaired neuronal function and enhance cell survival (Hayakawa et al., 2016). Conversely, damaged neurons can transfer dysfunctional mitochondria to adjacent astrocytes for degradation and clearance, suggesting that astrocytes not only provide energetic support but may also play an important role in intercellular mitochondrial quality control (Davis et al., 2014). Under pathological conditions such as chemotherapy-related neurotoxicity, astrocyte-mediated mitochondrial transfer is likewise considered neuroprotective and may, to some extent, alleviate mitochondrial damage and disturbances in energy metabolism (English et al., 2020).

**Figure 2:**
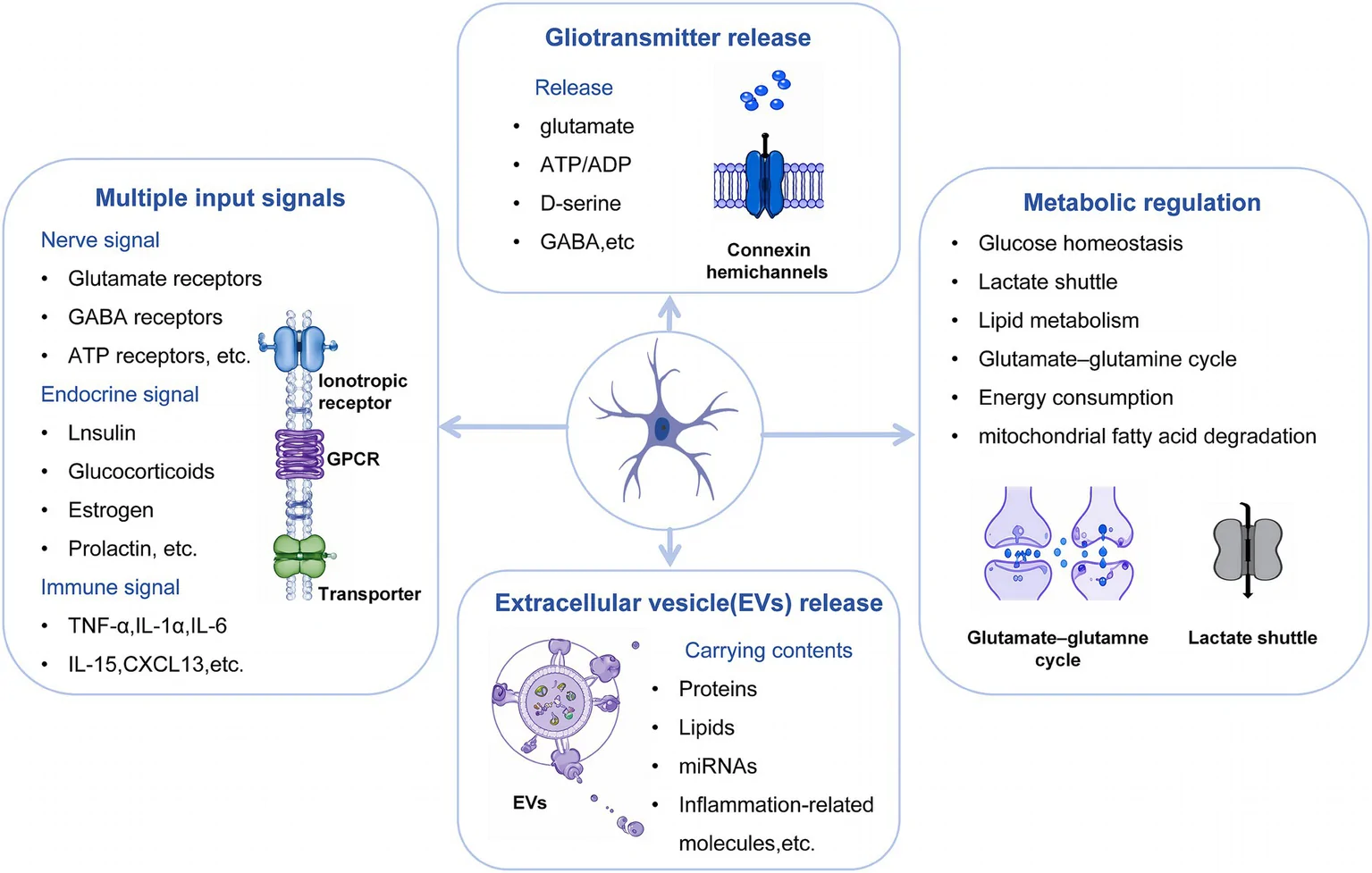
Information sensing and output of astrocytes. The diagram summarizes the major input signals sensed by astrocytes and their principal signaling, secretory, and metabolic outputs. The central astrocyte represents the integrative role of astrocytes in receiving diverse signals and coordinating downstream responses. Blue headings and arrows indicate the major functional categories. The receptor, transporter, channel, vesicle, and metabolic-cycle illustrations represent representative molecular structures or processes and are not intended to indicate exact molecular dimensions or stoichiometry. The arrows denote functional associations and output directions rather than a single linear signaling pathway. ADP, adenosine diphosphate; ATP, adenosine triphosphate; EVs, extracellular vesicles; GABA, γ-aminobutyric acid; GPCR, G-protein-coupled receptor.

In summary, astrocytic information output takes multiple forms, including transmitter-based, vesicle-mediated, inflammatory mediator-based, and metabolic modes. Through these diverse output mechanisms, astrocytes can respond to local neural activity, changes in hormone levels, and inflammatory signals, and can further influence the functional state of the NEI network (Information sensing and output of astrocytes in Figure 2).

### 4.3 Interactions between astrocytes and neurons, glial cells, the lymphoid-like system, and endocrine axes

Astrocytes are able to serve as key hubs within the NEI network not only because they possess the capacity for signal sensing and information output, but also because they are located at the intersection of interactive networks linking neurons, glial cells, the glymphatic system, and endocrine axes. In other words, the hub function of astrocytes is not determined by a single function, but is instead built upon continuous, dynamic, and highly plastic interactions among astrocytes, neurons, endocrine cells, and immune cells (Herrera Moro Chao et al., 2022; Mederos et al., 2018; Perea et al., 2014; Jensen et al., 2013).

First, in neuron–astrocyte interactions, these two cell types constitute the fundamental unit for regulating the synaptic microenvironment. From a structural perspective, astrocytes exhibit highly complex morphological features. Their somata give rise to numerous fine processes that extensively ensheath neuronal cell bodies, axon terminals, and synaptic structures. At the same time, their perivascular endfeet are in close contact with capillaries, microvessels, and the pial surface, thereby establishing a highly coupled structural framework among neurons, blood vessels, and the extracellular environment (Tewari et al., 2024; Ngoc et al., 2025; Durkee and Araque, 2019). Relying on this unique morphological organization, astrocytes can continuously sense changes in local neural activity and promptly regulate extracellular ionic homeostasis, neurotransmitter concentrations, and local metabolic states. On the one hand, astrocytes can clear neurotransmitters from the synaptic cleft, thereby preventing excessive excitatory transmission; on the other hand, they can participate in synaptic transmission and neural circuit regulation through the release of gliotransmitters, thus actively influencing the activity patterns and plasticity of neural networks (Durkee and Araque, 2019). Therefore, astrocytes are not merely supportive cells that passively ensheath neurons; rather, on the basis of their structural support, they actively regulate neural information transmission and modulation. At the same time, this regulation is not unidirectional. Changes in neuronal activity can, in turn, shape astrocytic phenotypes. For example, alterations in neurotransmitter release, neuropeptide signaling, and the rhythms of neuronal electrical activity can all influence astrocytic Ca^2+^ signaling, gene transcription, and energy metabolism (Petroccione et al., 2020; Zhou et al., 2022; Rupprecht et al., 2024). Thus, neurons and astrocytes form a highly coupled, mutually feedback-regulated dynamic regulatory loop.

Astrocyte–microglia interactions constitute an important basis for the regulation of immune homeostasis in the central nervous system. Studies have shown that, under conditions of inflammation, injury, or neurodegeneration, microglia can release inflammatory mediators such as TNF-α and IL-1α, thereby inducing astrocytes to transition toward reactive and even neurotoxic phenotypes (Liddelow et al., 2017; Habbas et al., 2015; Patani et al., 2023). This transition not only alters the structural and molecular characteristics of astrocytes, but also markedly reshapes the ways in which they regulate neurons and the immune microenvironment. Conversely, astrocytes do not passively receive inflammatory signals from microglia. Instead, they can reciprocally regulate microglial activation, migratory capacity, phagocytic function, and inflammatory responses through multiple molecules, including ATP, IL-15, and C-C motif chemokine ligand 2(CCL2) (Chen et al., 2024; Shaw et al., 2026; Rong et al., 2021). For example, astrocyte-derived IL-15 can enhance local immune-inflammatory responses and exacerbate brain tissue damage after ischemic brain injury. In addition, astrocytic IL-15 expression is upregulated during Alzheimer’s disease-related neuroinflammation, suggesting that it may participate in regulating the central inflammatory microenvironment (Li et al., 2017; Shaw et al., 2026). Moreover, CCL2 encapsulated in astrocyte-derived extracellular vesicles can promote microglial activation and induce neuronal apoptosis (Rong et al., 2021). Furthermore, astrocytes can prevent the development of excessive microglial activation through mechanisms such as interleukin-10 (IL-10), nuclear factor erythroid 2-related factor 2 (Nrf2)-mediated antioxidant responses, and bone morphogenetic protein (BMP) signaling regulation, thereby limiting the spread of inflammation and promoting the restoration of tissue homeostasis (Recasens et al., 2019; Oksanen et al., 2020; Li et al., 2025). In addition to interacting with microglia, astrocytes themselves possess distinct and active immunoregulatory capacities. They not only participate in maintaining immune homeostasis in the central nervous system, but also rapidly enter a reactive state under disease-related stress conditions, such as inflammation, ischemic injury, and neurodegenerative disorders. In this state, astrocytes can influence the functional status of neurons, microglia, and peripheral immune cells by secreting cytokines, chemokines, lipid mediators, and metabolic signals (Ziff et al., 2022; Lindblad et al., 2023; Shen et al., 2021; Sanmarco et al., 2021; Xing et al., 2018). Multi-omics studies have further shown that, under pathological conditions, astrocytes exhibit prominent features related to inflammation-associated pathways, reactive phenotypes, and pathological remodeling (Lindblad et al., 2023). Meanwhile, functional interactions between immune cells and astrocytes have also been demonstrated to play important roles in the onset of neuroinflammation, tissue repair, and disease progression (Sanmarco et al., 2021). Taken together, these findings indicate that astrocytes are not merely executors in neuroinflammation, but rather key regulatory nodes with dual roles in both amplifying and buffering inflammatory responses (Stephenson et al., 2018). Therefore, astrocytes are not only regulators of neural activity, but also important participants in central immune responses.

In addition, bidirectional interactions between astrocytes and oligodendrocytes are critical for maintaining the structural and functional homeostasis of central myelin (Nave and Werner, 2014; Kıray et al., 2016). Oligodendrocytes and their precursor cells, oligodendrocyte precursor cells (OPCs), are responsible for central myelin formation, insulation of axonal electrical signaling, and axonal energy support. The directed migration, proliferation, terminal differentiation of OPCs, as well as the survival of mature oligodendrocytes, are all highly dependent on astrocytes (Kıray et al., 2016; Moore et al., 2011). A series of studies has demonstrated that astrocytes can secrete a variety of soluble signaling molecules while also regulating the remodeling of extracellular matrix components, thereby modulating the entire process of OPCs development and participating in remyelination and white matter repair after demyelinating injury (Kıray et al., 2016; Nash et al., 2011; Moore et al., 2011). At the metabolic level, astrocytes rely on glutamate transport systems to maintain glutamate homeostasis in synaptic and white matter compartments. They continuously synthesize and release high-energy metabolic substrates such as lactate, while coordinately regulating local ionic balance and redox status. These functions help meet the high energetic demands required for myelin synthesis and membrane structural maintenance in oligodendrocytes, thereby ensuring the long-term stability of myelin (Nave and Werner, 2014; Cambron et al., 2012). Astrocytic regulation of myelin repair exhibits marked functional duality, as reactive astrocytes induced by pathological stimuli can produce diametrically opposite effects (Nash et al., 2011). Chronically and persistently activated astrocytes can release large amounts of proinflammatory cytokines, block OPCs migration and differentiation toward lesion sites, and inhibit remyelination. In contrast, moderately activated astrocytes during the early phase of injury can secrete abundant trophic and repair-associated mediators, improve the local microenvironment, increase the survival of oligodendrocyte lineage cells, and promote white matter repair (Nash et al., 2011; Moore et al., 2011). Together, these findings indicate that the regulatory roles of astrocytes are not limited to synaptic plasticity and local immune homeostasis in gray matter regions, but also extend to the development of white matter myelin, axonal energy metabolism, and the maintenance of white matter functional homeostasis.

Beyond direct intercellular interactions, astrocytes also influence the clearance of metabolic waste, interstitial fluid flow, and homeostasis of the neuroimmune microenvironment by participating in the formation and regulation of the glymphatic system, thereby further expanding their integrative role within the NEI network (Iliff et al., 2012; Nedergaard, 2013; Plog and Nedergaard, 2018). The glymphatic system is a central fluid circulation and clearance network composed of perivascular spaces, exchange pathways between cerebrospinal fluid and brain interstitial fluid, and astrocytic perivascular endfeet. The efficient operation of this system depends on the polarized distribution of the water channel aquaporin-4 (AQP4) on the surface of perivascular astrocytic endfeet (Iliff et al., 2012; Plog and Nedergaard, 2018). Studies have demonstrated that polarized AQP4 in astrocytic endfeet mediates transmembrane water transport, driving cerebrospinal fluid to penetrate deep into the brain parenchyma along periarterial perivascular spaces, while also facilitating the return and clearance of interstitial fluid carrying soluble metabolic waste through perivenous pathways (Iliff et al., 2012; Nedergaard, 2013). Under pathological conditions, reactive activation of astrocytes can disrupt the structural integrity of perivascular endfeet and lead to the loss of polarized AQP4 distribution, which directly impairs the efficiency of glymphatic fluid transport. This impairment may subsequently result in the persistent accumulation of toxic proteins, such as amyloid-β (Aβ), and proinflammatory mediators within the brain parenchyma, disturb the neuronal survival microenvironment, and contribute to a pathological process characterized by metabolic dysregulation and amplification of neuroinflammation (Nedergaard, 2013; Plog and Nedergaard, 2018). Taken together, these findings indicate that the regulatory functions of astrocytes are not limited to synaptic signaling and neuroimmune responses, but also link neurons, cerebral vasculature, and the brain’s metabolic waste clearance system.

Interactions between astrocytes and endocrine axes are primarily centered in the hypothalamus and related neuroendocrine nuclei. The hypothalamus is a key region for integrating peripheral metabolic information with central neural regulation. It not only mediates endocrine regulation, but also serves as an important center for stress responses and energy homeostasis. In these regions, astrocytes can integrate hormonal signals, nutritional status, and neural activity inputs, and participate in the fine regulation of the hypothalamic–pituitary–adrenal (HPA) axis, the hypothalamic–pituitary–thyroid (HPT) axis, and energy homeostasis networks by modulating the excitability of releasing hormone neurons (Herrera Moro Chao et al., 2022; Schouten et al., 2025; Castillo et al., 2024; Clasadonte and Prevot, 2018). In the hypothalamus, astrocytes are closely associated with neurosecretory neurons and surrounding vascular structures, allowing them to directly sense diverse signals and thereby participate in the regulation of energy expenditure, stress responses, and endocrine homeostasis. In addition, glucocorticoid-, estrogen-, and prolactin-related signals can influence astrocytic reactivity, metabolic status, and secretory profiles. Conversely, astrocytes can regulate the activity of hypothalamic neuroendocrine neurons by modulating neurotransmitter clearance efficiency, altering the local ionic environment, and releasing gliotransmitters (Lu et al., 2020; Castillo et al., 2024; Lai et al., 2024; Maatouk et al., 2019). Studies have shown that astrocytes can also affect neuronal excitability, synaptic development, and intercellular communication through extracellular vesicles, neurotrophic factors, and other soluble mediators, further expanding their role in neuroendocrine regulation (Makrygianni and Chrousos, 2023; Kim et al., 2017). Thus, astrocytes are not merely supportive elements embedded within the neuroendocrine system, but important functional units involved in hormonal signal integration, regulation of neuronal output, and maintenance of homeostasis.

In summary, through interactions with neurons, glial cells, the glymphatic system, and endocrine axes, astrocytes regulate neural activity, immune responses, and endocrine homeostasis. Therefore, we propose that astrocytes represent important integrative nodes within the NEI network.

### 4.4 Reactive transformation of astrocytes and functional remodeling under disease conditions

Under normal physiological conditions, astrocytes help maintain central and systemic homeostasis by integrating neural, endocrine, and immune signals. However, when the body is exposed to disease, inflammation, or persistent stress, the phenotype and function of astrocytes undergo marked remodeling. Astrocytes then enter a reactive state and participate in pathological processes such as neuroinflammation, neurodegeneration, and tissue injury repair (Schober et al., 2022; Patani et al., 2023; Stephenson et al., 2018; Lawrence et al., 2023). Reactive astrocytes do not represent a single, static pathological phenotype; rather, they exhibit substantial heterogeneity and context dependence. Their molecular features, spatial distribution, and functions change dynamically according to disease type, disease stage, and the microenvironment of specific brain regions (Ziff et al., 2022; Lindblad et al., 2023; Shen et al., 2021; Sanmarco et al., 2021).

Across the disease spectrum, reactive changes in astrocytes are widely observed in neurological disorders such as stroke, Alzheimer’s disease, and Parkinson’s disease, and are often accompanied by enhanced inflammatory responses, abnormal intercellular crosstalk, and disrupted calcium signaling (Li et al., 2022; Huffels et al., 2023; Li et al., 2024; Kim et al., 2024; Huang et al., 2022). In Alzheimer’s disease, Aβ-associated pathology can markedly disrupt neuron–glia interactions and synaptic microenvironmental homeostasis. Astrocytes contribute to disease progression by participating in synaptic regulation, amplification of inflammation, and impairment of neuronal support functions. Moreover, studies have shown that aberrant activation of hippocampal astrocytes itself can induce neuroinflammation and lead to cognitive decline (Huffels et al., 2023; Kim et al., 2024). These findings further suggest that astrocytes are not only responders to the pathological environment, but may also be important contributors to the development of cognitive impairment. In Parkinson’s disease and related synucleinopathies, α-synuclein can induce transcriptional-state remodeling in astrocytes and mediate neurotoxicity through abnormal astrocyte–neuron crosstalk. In addition, the transfer of neuron-derived α-synuclein to astrocytes can further trigger neuroinflammatory responses and exacerbate blood–brain barrier damage, suggesting that astrocytes play an important role in linking the propagation of protein pathology with neurovascular unit dysfunction (Li et al., 2024; Huang et al., 2022). Taken together, these observations indicate that pathological remodeling of astrocytes is not limited to a single disease, but represents a shared mechanism across multiple classes of central nervous system disorders.

Mechanistically, functional remodeling of astrocytes under disease or stress conditions is first reflected by altered responsiveness to external signals, including heightened and potentially aberrantly amplified responses to neurotransmitters, inflammation-related signals, and hormonal regulation (Guttenplan et al., 2025; Li et al., 2020; Lai et al., 2024). Second, astrocytic functions may shift from homeostatic support toward inflammatory amplification or neurotoxic effects. For example, under pathological conditions such as cerebral ischemia, astrocytes can exacerbate neuroinflammation through C-C motif chemokine ligand 11(CCL11)-C-C motif chemokine receptor 3(CCR3)-related signaling; in models of Alzheimer’s disease, reactive astrocytes can also release extracellular vesicles with mitochondrial toxicity, thereby aggravating neuronal injury and promoting disease progression (Crivelli et al., 2023; Yin et al., 2026). In addition, pathological remodeling of astrocytes may enhance interactions between local central lesions and peripheral immune status by affecting the brain–immune interface, the perivascular microenvironment, and brain lymphatic-related clearance pathways, thereby amplifying NEI network imbalance on a broader scale (Li et al., 2025; Gao et al., 2023; Hasegawa-Ishii et al., 2016).

However, reactive astrocytes do not necessarily imply exacerbated injury. In contrast, at certain disease stages or under specific microenvironmental conditions, reactive astrocytes can exert protective effects. For instance, astrocytes can participate in tissue repair, synaptic reconstruction, and neural network recovery by enhancing antioxidant and immunomodulatory capacities, regulating local inflammatory responses, and releasing paracrine signals or extracellular vesicles that promote synaptogenesis (Oksanen et al., 2020; Patel and Weaver, 2021; Hwang and Bergmann, 2018). Therefore, reactive astrocytes should be regarded not simply as a harmful response, but rather as a dynamic adaptive process driven by pathological stimuli, precisely regulated by the spatiotemporal microenvironment, and characterized by stage-specific features. In addition, the previously proposed dichotomy of A1/A2 reactive astrocytes has provided an important framework for understanding astrocyte reactivity. In this model, A1 reactive astrocytes are generally associated with proinflammatory and neurotoxic effects, whereas A2 reactive astrocytes are more closely linked to repair and neuroprotection. Although this dichotomous model was broadly useful and conceptually informative in the early stages of reactive astrocyte research, its limitations have become increasingly evident with advances in single-cell transcriptomics, spatial omics, and dynamic disease-tracking studies. Recent studies have shown that astrocytes can undergo transitions among substates during neurodegenerative processes, and that distinct substates are closely associated with disease progression and the severity of neurodegenerative injury (Zhang et al., 2025). These findings suggest that changes in reactive astrocyte states do not occur as fixed categories with distinct functions, but are more likely to represent multiple continuously evolving and interconvertible states during disease progression. Thus, although the A1/A2 framework remains conceptually instructive, it is no longer sufficient to fully capture the true complexity of reactive astrocytes. Future studies should integrate time-series analyses, multi-omics approaches, and spatially resolved strategies to further elucidate the mechanisms underlying the formation and transition of astrocyte substates.

In summary, reactive remodeling of astrocytes under disease conditions represents an important component of NEI network imbalance. Further elucidation of the molecular characteristics, functions, and actionable targets of different reactive astrocyte subtypes will not only deepen our understanding of astrocyte heterogeneity, but also provide new theoretical foundations and strategic directions for precision treatment of NEI-related disorders.

## 5 Translational relevance and challenges of astrocyte-targeted interventions

As the hub-like role of astrocytes within the NEI network has become increasingly clear, their translational value as therapeutic targets has attracted growing attention. Astrocytes are the predominant glial cell type in the central nervous system. They are widely distributed across different brain regions and form close interactions with neurons, blood vessels, and other glial cells, thereby playing important roles in the regulation of synaptic function, maintenance of ionic and neurotransmitter homeostasis, support of energy metabolism, stabilization of the blood–brain barrier, and modulation of central immune and inflammatory responses (Escartin et al., 2021; Allen and Lyons, 2018). Recent studies have shown that astrocytes not only participate in the initiation and progression of pathological processes in various neurological disorders, but also influence multiple key events through transitions in reactive states, including amplification of local inflammation, impairment of neuronal function, and disruption of the neurovascular unit (Liddelow et al., 2017; Escartin et al., 2021). Therefore, astrocyte-targeted interventions are considered to have the potential advantage of simultaneously modulating multiple pathological processes, and astrocytes have gradually emerged as important therapeutic targets in neurological diseases such as stroke (Liu and Chopp, 2016).

Currently, astrocyte-targeted intervention strategies mainly focus on several aspects. First, regulating the state transitions of reactive astrocytes and their associated signaling pathways represents an important direction of current research. The Janus kinase/signal transducer and activator of transcription 3(JAK/STAT3) pathway plays a critical role in astrocyte activation, gliosis, and scar formation, and intervention in this pathway may help attenuate harmful inflammatory responses and aberrant tissue remodeling (Liddelow and Barres, 2017; Herrmann et al., 2008). Second, abnormal intercellular communication involving astrocytes is also an important therapeutic target. For example, modulation of gap junctions and connexins may reduce the abnormal release of signaling molecules such as ATP and glutamate, inhibit the spread of inflammatory signals, and alleviate neuronal injury mediated by aberrant glia–neuron signaling (Giaume et al., 2021). Studies have also indicated that improving astrocyte-associated metabolic abnormalities and brain clearance functions is of considerable importance. Such strategies include correcting imbalanced lactate metabolism, restoring astrocytic metabolic support for neurons, and maintaining the integrity of perivascular endfeet and AQP4 polarity, thereby promoting cerebrospinal fluid–interstitial fluid exchange and the clearance of metabolic waste (Magistretti and Allaman, 2018; Rasmussen et al., 2018). In addition, targeted gene delivery to specific cell types or brain regions within the central nervous system using vectors such as adeno-associated virus (AAV) provides new technical approaches for improving the selectivity and precision of astrocyte-directed interventions (Hudry and Vandenberghe, 2019). Meanwhile, stem cell- and regenerative medicine-based strategies are also being developed, including the use of induced pluripotent stem cell (iPSC)-derived astrocytes or related progenitor cells for disease modeling and potential cell replacement therapy, with the aim of reconstructing the damaged microenvironment, improving metabolic support, and promoting tissue repair (Sloan et al., 2017). Overall, astrocytes may represent a promising therapeutic target.

Although astrocyte-targeted therapy has substantial potential, its clinical translation still faces multiple challenges. First, astrocytes exhibit marked regional heterogeneity and functional diversity. Astrocytes in different brain regions differ in molecular expression, morphology, and functional state, making it difficult to treat them as a single target for simple activation or inhibition. Second, astrocyte-targeted therapy requires highly cell-selective delivery systems. AAV-mediated gene delivery has become an important technology for nervous system therapies; however, its targeting efficiency and safety for astrocytes in specific brain regions and under pathological conditions still need to be further improved (Hudry and Vandenberghe, 2019). In addition, most existing studies rely on rodent models, whereas human astrocytes differ substantially from mouse astrocytes in morphological complexity, gene expression, and functional characteristics, which limits the direct extrapolation of animal experimental findings to the clinic (Oberheim et al., 2009; Zhang et al., 2016). Human iPSC and brain organoid technologies provide new approaches to addressing this issue and can be used to model human astrocyte maturation, establish disease models, and perform drug screening (Sloan et al., 2017; Qian et al., 2019). Future studies should integrate human-derived models, brain organoids, single-cell omics, and precision delivery technologies to establish brain region-specific and disease-specific astrocyte intervention strategies.

## 6 Discussion

Taken together, existing evidence indicates that astrocytes are no longer viewed merely as supportive cells in the traditional sense, but are increasingly recognized as important glial cells involved in neural, endocrine, and immune regulation. It should be emphasized that the conceptual framework proposed in this review, which regards astrocytes as integrative nodes in the NEI network, does not simply refer to their separate involvement in neural, endocrine, and immune processes. Rather, it further suggests that astrocytes may be positioned at the convergence of multiple sources of signaling and may possess the potential capacity for cross-system information integration and regulation. Compared with studies focused on a single signaling axis, the significance of this framework lies in its attempt to explain how astrocytes coordinate changes in the local microenvironment under different physiological and pathological conditions and subsequently influence homeostasis. However, it must be acknowledged that this framework is currently based primarily on the integration of evidence across fields and mechanistic inference, and direct evidence demonstrating, within the same experimental system, that astrocytes can sense, integrate, and output neural, endocrine, and immune signals is still lacking.

At present, this field continues to face substantial heterogeneity in evidence sources and a lack of unified research paradigms. Previous studies have employed a variety of approaches, including primary cell culture, transgenic animals, disease animal models, and multi-omics analyses. However, these models differ markedly in astrocyte maturity, cellular environment, and species origin, which may affect the comparability of findings. For example, human astrocytes are not fully equivalent to rodent astrocytes in terms of morphological complexity, molecular characteristics, and functional maturity. In addition, astrocytes in different brain regions, as well as those located in white matter versus gray matter environments, differ in gene expression and function. It is particularly important to note that many current studies still focus primarily on a single disease model, a single brain region, or a single stimulus condition, making it difficult to comprehensively capture the simultaneous sensing and integration of neural, immune, and endocrine signals by astrocytes under authentic physiological and pathological states. Moreover, mature experimental strategies and evaluation systems are still lacking for linking local functional changes in astrocytes to organism-level neuro–endocrine–immune regulation. Therefore, future studies should further standardize and integrate model selection, experimental design, and analytical methods to improve the reliability and translational value of relevant conclusions.

Future research should proceed in several directions. First, single-cell omics, spatial omics, multimodal *in vivo* imaging, and functional manipulation technologies should be integrated to establish a chain of evidence spanning molecular, cellular, tissue, and organismal levels. Second, comparative studies across brain regions, diseases, and models should be strengthened to distinguish general mechanisms from context-specific mechanisms. Finally, greater emphasis should be placed on validation using human-derived cellular models, brain organoids, and clinical samples, in order to enhance the translatability of related findings.

## Glossary

NEI: Neuro-endocrine-immune
IL-1α: Interleukin-1α
TNF: Tumor necrosis factor
C1q: Complement component 1q
MAFG: MAF bZIP transcription factor G
ATP: Adenosine triphosphate
GABA: γ-aminobutyric acid
GPCR: G protein-coupled receptor
TNF-α: Tumor necrosis factor alpha
CXCL13: C-X-C motif chemokine ligand 13
IL-6: Interleukin-6
IL-15: Interleukin-15
PGE2: Prostaglandin E2
Cx43: Connexin 43
EVs: Extracellular vesicles
CCL2: C-C motif chemokine ligand 2
Nrf2: Nuclear factor erythroid 2–related factor 2
BMP: Bone morphogenetic protein
OPCs: Oligodendrocyte precursor cells
AQP4: Aquaporin-4
Aβ: Amyloid-β
HPA: Hypothalamic–pituitary–adrenal
HPT: Hypothalamic–pituitary–thyroid
CCR3: C-C motif chemokine receptor 3
JAK/STAT3: Janus kinase/signal transducer and activator of transcription 3
AAV: Adeno-associated virus
iPSC: Induced pluripotent stem cell

## References

[ref1] Allen N. J. Lyons D. A. (2018). Glia as architects of central nervous system formation and function. Science 362, 181–185. doi: 10.1126/science.aat0473, 30309945 PMC6292669

[ref2] Alvarez J. I. Dodelet-Devillers A. Kebir H. Ifergan I. Fabre P. J. Terouz S. . (2011). The hedgehog pathway promotes blood-brain barrier integrity and CNS immune quiescence. Science 334, 1727–1731. doi: 10.1126/science.1206936, 22144466

[ref3] Ameroso D. Rios M. (2024). Synaptic plasticity and the role of astrocytes in central metabolic circuits. WIREs Mechanisms Dis. 16:e1632. doi: 10.1002/wsbm.1632, 37833830 PMC10842964

[ref4] Bartels T. Rowitch D. H. Bayraktar O. A. (2024). Generation of mammalian astrocyte functional heterogeneity. Cold Spring Harb. Perspect. Biol. 16:a041351. doi: 10.1101/cshperspect.a041351, 38692833 PMC11529848

[ref5] Ben Haim L. Rowitch D. H. (2016). Functional diversity of astrocytes in neural circuit regulation. Nat. Rev. Neurosci. 18, 31–41. doi: 10.1038/nrn.2016.159, 27904142

[ref6] Bocchi R. Thorwirth M. Simon-Ebert T. Koupourtidou C. Clavreul S. Kolf K. . (2025). Astrocyte heterogeneity reveals region-specific astrogenesis in the white matter. Nat. Neurosci. 28, 457–469. doi: 10.1038/s41593-025-01878-6, 39994409 PMC11893471

[ref7] Bonvento G. Bolaños J. P. (2021). Astrocyte-neuron metabolic cooperation shapes brain activity. Cell Metab. 33, 1546–1564. doi: 10.1016/j.cmet.2021.07.006, 34348099

[ref8] Bugiani M. Plug B. C. Man J. H. K. Breur M. van der Knaap M. S. (2022). Heterogeneity of white matter astrocytes in the human brain. Acta Neuropathol. 143, 159–177. doi: 10.1007/s00401-021-02391-3, 34878591

[ref9] Cambron M. D'Haeseleer M. Laureys G. Clinckers R. Debruyne J. De Keyser J. (2012). White-matter astrocytes, axonal energy metabolism, and axonal degeneration in multiple sclerosis. J. Cereb. Blood Flow Metab. 32, 413–424. doi: 10.1038/jcbfm.2011.193, 22214904 PMC3293127

[ref10] Castillo X. Ortiz G. Arnold E. Wu Z. Romo T. Y. Clapp C. . (2024). The influence of the prolactin/vasoinhibin axis on post-stroke lesion volume, astrogliosis, and survival. J. Neuroendocrinol. 36:e13415. doi: 10.1111/jne.13415, 38808481

[ref11] Chai H. Diaz-Castro B. Shigetomi E. Monte E. Octeau J. C. Yu X. . (2017). Neural circuit-specialized astrocytes: transcriptomic, proteomic, morphological, and functional evidence. Neuron 95, 531–549.e9. doi: 10.1016/j.neuron.2017.06.029, 28712653 PMC5811312

[ref12] Chen Y. L. Feng X. L. Cheung C. W. Liu J. A. (2022). Mode of action of astrocytes in pain: from the spinal cord to the brain. Prog. Neurobiol. 219:102365. doi: 10.1016/j.pneurobio.2022.102365, 36228888

[ref13] Chen Y. Luan P. Liu J. Wei Y. Wang C. Wu R. . (2024). Spatiotemporally selective astrocytic ATP dynamics encode injury information sensed by microglia following brain injury in mice. Nat. Neurosci. 27, 1522–1533. doi: 10.1038/s41593-024-01680-w, 38862791

[ref14] Chen Y. Ye Y. Jia J. Long B. Dou T. Yan X. (2025). Advances in astrocytic calcium signaling research. Front. Cell. Neurosci. 19:1719532. doi: 10.3389/fncel.2025.1719532, 41383745 PMC12689559

[ref15] Clasadonte J. Prevot V. (2018). The special relationship: glia-neuron interactions in the neuroendocrine hypothalamus. Nat. Rev. Endocrinol. 14, 25–44. doi: 10.1038/nrendo.2017.124, 29076504

[ref16] Codeluppi S. Fernandez-Zafra T. Sandor K. Kjell J. Liu Q. Abrams M. . (2014). Interleukin-6 secretion by astrocytes is dynamically regulated by PI3K-mTOR-calcium signaling. PLoS One 9:e92649. doi: 10.1371/journal.pone.0092649, 24667246 PMC3965459

[ref17] Cohen-Salmon M. Guille N. Boulay A.-C. (2025). Development of perivascular astrocyte processes. Front. Neurosci. 19:1585340. doi: 10.3389/fnins.2025.1585340, 40688418 PMC12271147

[ref18] Crivelli S. M. Quadri Z. Vekaria H. J. Zhu Z. Tripathi P. Elsherbini A. . (2023). Inhibition of acid sphingomyelinase reduces reactive astrocyte secretion of mitotoxic extracellular vesicles and improves Alzheimer’s disease pathology in the 5xFAD mouse. Acta Neuropathol. Commun. 11:135. doi: 10.1186/s40478-023-01633-7, 37605262 PMC10440899

[ref19] Cuellar-Santoyo A. O. Ruiz-Rodríguez V. M. Mares-Barbosa T. B. Patrón-Soberano A. Howe A. G. Portales-Pérez D. P. . (2022). Revealing the contribution of astrocytes to glutamatergic neuronal transmission. Front. Cell. Neurosci. 16:1037641. doi: 10.3389/fncel.2022.1037641, 36744061 PMC9893894

[ref20] Datta Chaudhuri A. Dasgheyb R. M. DeVine L. R. Bi H. Cole R. N. Haughey N. J. (2020). Stimulus-dependent modifications in astrocyte-derived extracellular vesicle cargo regulate neuronal excitability. Glia 68, 128–144. doi: 10.1002/glia.23708, 31469478

[ref21] Davalos D. Grutzendler J. Yang G. Kim J. V. Zuo Y. Jung S. . (2005). ATP mediates rapid microglial response to local brain injury in vivo. Nat. Neurosci. 8, 752–758. doi: 10.1038/nn1472, 15895084

[ref22] Davis C. H. Kim K. Y. Bushong E. A. Mills E. A. Boassa D. Kinebuchi M. . (2014). Transcellular degradation of axonal mitochondria. Proc. Natl. Acad. Sci. USA 111, 9633–9638. doi: 10.1073/pnas.1404651111, 24979790 PMC4084443

[ref23] Djukic B. Casper K. B. Philpot B. D. Chin L. S. McCarthy K. D. (2007). Conditional knock-out of Kir4.1 leads to glial membrane depolarization, inhibition of potassium and glutamate uptake, and enhanced short-term synaptic potentiation. J. Neurosci. 27, 11354–11365. doi: 10.1523/JNEUROSCI.0723-07.2007, 17942730 PMC6673037

[ref24] Durkee C. A. Araque A. (2019). Diversity and specificity of astrocyte-neuron communication. Neuroscience 396, 73–78. doi: 10.1016/j.neuroscience.2018.11.010, 30458223 PMC6494094

[ref25] Endo F. Kasai A. Soto J. S. Yu X. Qu Z. Hashimoto H. . (2022). Molecular basis of astrocyte diversity and morphology across the CNS in health and disease. Science 378:eadc9020. doi: 10.1126/science.adc9020, 36378959 PMC9873482

[ref26] English K. Shepherd A. Uzor N. E. Trinh R. Kavelaars A. Heijnen C. J. (2020). Astrocytes rescue neuronal health after cisplatin treatment through mitochondrial transfer. Acta Neuropathol. Commun. 8:36. doi: 10.1186/s40478-020-00897-7, 32197663 PMC7082981

[ref27] Escartin C. Galea E. O'Callaghan J. P. Serrano-Pozo A. Steinhäuser C. Volterra A. . (2021). Reactive astrocyte nomenclature, definitions, and future directions. Nat. Neurosci. 24, 312–325. doi: 10.1038/s41593-020-00783-4, 33589835 PMC8007081

[ref28] Ficker C. Rozmer K. Kató E. Andó R. D. Schumann L. Krügel U. . (2014). Astrocyte-neuron interaction in the substantia gelatinosa of the spinal cord dorsal horn via P2X7 receptor-mediated release of glutamate and reactive oxygen species. Glia 62, 1671–1686. doi: 10.1002/glia.22707, 24895290

[ref29] Gao L. Pan X. Zhang J. H. Xia Y. (2023). Glial cells: an important switch for the vascular function of the central nervous system. Front. Cell. Neurosci. 17:1166770. doi: 10.3389/fncel.2023.1166770, 37206667 PMC10188976

[ref30] García-Cáceres C. Quarta C. Gruber T. Legutko B. Jastroch M. Le Thuc O. . (2016). Astrocytic insulin signaling couples brain glucose uptake with nutrient availability. Cell 166, 867–880. doi: 10.1016/j.cell.2016.07.028, 27518562 PMC8961449

[ref31] Giaume C. Naus C. C. Sáez J. C. Leybaert L. (2021). Glial connexins and pannexins in the healthy and diseased brain. Physiol. Rev. 101, 93–145. doi: 10.1152/physrev.00043.2018, 32326824

[ref32] Guttenplan K. A. Maxwell I. Santos E. Borchardt L. A. Manzo E. Abalde-Atristain L. . (2025). GPCR signaling gates astrocyte responsiveness to neurotransmitters and control of neuronal activity. Science 388, 763–768. doi: 10.1126/science.adq5729, 40373148 PMC13132101

[ref33] Habbas S. Santello M. Becker D. Stubbe H. Zappia G. Liaudet N. . (2015). Neuroinflammatory TNFα impairs memory via astrocyte signaling. Cell 163, 1730–1741. doi: 10.1016/j.cell.2015.11.023, 26686654

[ref34] Hasegawa-Ishii S. Inaba M. Umegaki H. Unno K. Wakabayashi K. Shimada A. (2016). Endotoxemia-induced cytokine-mediated responses of hippocampal astrocytes transmitted by cells of the brain-immune interface. Sci. Rep. 6:25457. doi: 10.1038/srep25457, 27149601 PMC4857737

[ref35] Hayakawa K. Esposito E. Wang X. Terasaki Y. Liu Y. Xing C. . (2016). Transfer of mitochondria from astrocytes to neurons after stroke. Nature 535, 551–555. doi: 10.1038/nature18928, 27466127 PMC4968589

[ref36] Hennes M. Richter M. L. Fischer-Sternjak J. Götz M. (2025). Astrocyte diversity and subtypes: aligning transcriptomics with multimodal perspectives. EMBO Rep. 26, 4203–4218. doi: 10.1038/s44319-025-00529-y, 40750713 PMC12420811

[ref37] Herrera Moro Chao D. Pham C. Castel J. Morel C. Hassouna R. Mouffle C. . (2022). Hypothalamic astrocytes control systemic glucose metabolism and energy balance. Cell Metab. 34, 1532–1547.e6. doi: 10.1016/j.cmet.2022.09.002, 36198294 PMC9615252

[ref38] Herrmann J. E. Imura T. Qi J. Ao Y. Nguyen T. K. Korsak R. A. . (2008). STAT3 is a critical regulator of astrogliosis and scar formation after spinal cord injury. J. Neurosci. 28, 7231–7243. doi: 10.1523/JNEUROSCI.1709-08.2008, 18614693 PMC2583788

[ref39] Huang J. Ding J. Wang X. Gu C. He Y. Li Y. . (2022). Transfer of neuron-derived α-synuclein to astrocytes induces neuroinflammation and blood-brain barrier damage after methamphetamine exposure: involving the regulation of nuclear receptor-associated protein 1. Brain Behav. Immun. 106, 247–261. doi: 10.1016/j.bbi.2022.09.002, 36089218

[ref40] Hudry E. Vandenberghe L. H. (2019). Therapeutic AAV gene transfer to the nervous system: a clinical reality. Neuron 101, 839–862. doi: 10.1016/j.neuron.2019.02.017, 30844402 PMC11804970

[ref41] Huffels C. F. M. Middeldorp J. Hol E. M. (2023). Aβ pathology and neuron-glia interactions: a synaptocentric view. Neurochem. Res. 48, 1026–1046. doi: 10.1007/s11064-022-03699-6, 35976488 PMC10030451

[ref42] Hwang M. Bergmann C. C. (2018). Alpha/beta interferon (IFN-α/β) signaling in astrocytes mediates protection against viral encephalomyelitis and regulates IFN-γ-dependent responses. J. Virol. 92, e01901–e01917. doi: 10.1128/JVI.01901-17, 29491163 PMC5923078

[ref43] Iliff J. J. Wang M. Liao Y. Plogg B. A. Peng W. Gundersen G. A. . (2012). A paravascular pathway facilitates CSF flow through the brain parenchyma and the clearance of interstitial solutes, including amyloid β. Sci. Transl. Med. 4:147ra111. doi: 10.1126/scitranslmed.3003748, 22896675 PMC3551275

[ref44] Illes P. Burnstock G. Tang Y. (2019). Astroglia-derived ATP modulates CNS neuronal circuits. Trends Neurosci. 42, 885–898. doi: 10.1016/j.tins.2019.09.006, 31704181

[ref45] Jensen C. J. Massie A. De Keyser J. (2013). Immune players in the CNS: the astrocyte. J. Neuroimmune Pharmacol. 8, 824–839. doi: 10.1007/s11481-013-9480-6, 23821340

[ref46] Jiang B. C. Cao D. L. Zhang X. Zhang Z. J. He L. N. Li C. H. . (2016). CXCL13 drives spinal astrocyte activation and neuropathic pain via CXCR5. J. Clin. Invest. 126, 745–761. doi: 10.1172/JCI81950, 26752644 PMC4731172

[ref47] Karpf J. Unichenko P. Chalmers N. Beyer F. Wittmann M. T. Schneider J. . (2022). Dentate gyrus astrocytes exhibit layer-specific molecular, morphological and physiological features. Nat. Neurosci. 25, 1626–1638. doi: 10.1038/s41593-022-01192-5, 36443610

[ref48] Kim J. H. Michiko N. Choi I. S. Kim Y. Jang I. S. Suk K. . (2024). Aberrant activation of hippocampal astrocytes causes neuroinflammation and cognitive decline in mice. PLoS Biol. 22:e3002687. doi: 10.1371/journal.pbio.3002687, 38991663 PMC11239238

[ref49] Kim Y. Park S. J. Chen Y. M. (2017). Mesencephalic astrocyte-derived neurotrophic factor (MANF), a new player in endoplasmic reticulum diseases: structure, biology, and therapeutic roles. Transl. Res. 188, 1–9. doi: 10.1016/j.trsl.2017.06.010, 28719799 PMC5601018

[ref50] Kıray H. Lindsay S. L. Hosseinzadeh S. Barnett S. C. (2016). The multifaceted role of astrocytes in regulating myelination. Exp. Neurol. 283, 541–549. doi: 10.1016/j.expneurol.2016.03.009, 26988764 PMC5019113

[ref51] Kwon W. Williamson M. R. Deneen B. (2025). A functional perspective on astrocyte heterogeneity. Trends Neurosci. 48, 691–705. doi: 10.1016/j.tins.2025.06.009, 40695641 PMC12313154

[ref52] Lai W. Huang S. Liu J. Zhou B. Yu Z. Brown J. . (2024). Toll-like receptor 4-dependent innate immune responses are mediated by intracrine corticosteroids and activation of glycogen synthase kinase-3β in astrocytes. FASEB J. 38:e23781. doi: 10.1096/fj.202301923RR, 38941212

[ref53] Lawrence J. M. Schardien K. Wigdahl B. Nonnemacher M. R. (2023). Roles of neuropathology-associated reactive astrocytes: a systematic review. Acta Neuropathol. Commun. 11:42. doi: 10.1186/s40478-023-01526-9, 36915214 PMC10009953

[ref54] Li Y. Hao J. Wang W. Su Z. Tian X. Wang H. . (2025). Inhibition of astrocyte BMP signaling alleviates neuroinflammation in experimental models of Parkinson’s disease. Cell Death Discov. 11:528. doi: 10.1038/s41420-025-02812-2, 41213924 PMC12603212

[ref55] Li M. Li Z. Yao Y. Jin W. N. Wood K. Liu Q. . (2017). Astrocyte-derived interleukin-15 exacerbates ischemic brain injury via propagation of cellular immunity. Proc. Natl. Acad. Sci. USA 114, E396–E405. doi: 10.1073/pnas.1612930114, 27994144 PMC5255606

[ref56] Li K. Ling H. Huang W. Luo W. Gu C. Tao B. . (2024). Single-cell RNA-sequencing analysis reveals α-syn induced astrocyte-neuron crosstalk-mediated neurotoxicity. Int. Immunopharmacol. 139:112676. doi: 10.1016/j.intimp.2024.112676, 39053230

[ref57] Li J. Liu M. J. Du W. J. Peng X. L. Deng H. Zi H. X. . (2025). Neural-activity-regulated and glia-mediated control of brain lymphatic development. Cell 188, 3274–3290.e16. doi: 10.1016/j.cell.2025.04.008, 40311620

[ref58] Li D. Liu X. Liu T. Liu H. Tong L. Jia S. . (2020). Neurochemical regulation of the expression and function of glial fibrillary acidic protein in astrocytes. Glia 68, 878–897. doi: 10.1002/glia.23734, 31626364

[ref59] Li H. Zhao Y. Dai R. Geng P. Weng D. Wu W. . (2025). Astrocytes release ATP/ADP and glutamate in flashes via vesicular exocytosis. Mol. Psychiatry 30, 2475–2489. doi: 10.1038/s41380-024-02851-8, 39578520

[ref60] Li L. Zhou J. Han L. Wu X. Shi Y. Cui W. . (2022). The specific role of reactive astrocytes in stroke. Front. Cell. Neurosci. 16:850866. doi: 10.3389/fncel.2022.850866, 35321205 PMC8934938

[ref61] Liddelow S. A. Barres B. A. (2017). Reactive astrocytes: production, function, and therapeutic potential. Immunity 46, 957–967. doi: 10.1016/j.immuni.2017.06.006, 28636962

[ref62] Liddelow S. A. Guttenplan K. A. Bennett F. C. Münch A. E. Wilton D. K. Napier B. A. . (2017). Neurotoxic reactive astrocytes are induced by activated microglia. Nature 541, 481–487. doi: 10.1038/nature21029, 28099414 PMC5404890

[ref63] Lindblad C. Neumann S. Kolbeinsdóttir S. Zachariadis V. Thelin E. P. Enge M. . (2023). Stem cell-derived brainstem mouse astrocytes obtain a neurotoxic phenotype in vitro upon neuroinflammation. J. Inflamm. 20:22. doi: 10.1186/s12950-023-00349-8, 37370141 PMC10303821

[ref64] Liu Z. Chopp M. (2016). Astrocytes, therapeutic targets for neuroprotection and neurorestoration in ischemic stroke. Prog. Neurobiol. 144, 103–120. doi: 10.1016/j.pneurobio.2015.09.008, 26455456 PMC4826643

[ref65] Liu J. Feng X. Wang Y. Xia X. Zheng J. C. (2022). Astrocytes: GABAceptive and GABAergic cells in the brain. Front. Cell. Neurosci. 16:892497. doi: 10.3389/fncel.2022.892497, 35755777 PMC9231434

[ref66] Lu Y. Sareddy G. R. Wang J. Zhang Q. Tang F. L. Pratap U. P. . (2020). Neuron-derived estrogen is critical for astrocyte activation and neuroprotection of the ischemic brain. J. Neurosci. 40, 7355–7374. doi: 10.1523/JNEUROSCI.0115-20.2020, 32817249 PMC7534920

[ref67] Maatouk L. Yi C. Carrillo-de Sauvage M. A. Compagnion A. C. Hunot S. Ezan P. . (2019). Glucocorticoid receptor in astrocytes regulates midbrain dopamine neurodegeneration through connexin hemichannel activity. Cell Death Different. 26, 580–596. doi: 10.1038/s41418-018-0150-3, 30006609 PMC6370798

[ref68] MacDonald A. J. Robb J. L. Morrissey N. A. Beall C. Ellacott K. L. J. (2019). Astrocytes in neuroendocrine systems: an overview. J. Neuroendocrinol. 31:e12726. doi: 10.1111/jne.12726, 31050045

[ref69] Magistretti P. J. Allaman I. (2018). Lactate in the brain: from metabolic end-product to signalling molecule. Nat. Rev. Neurosci. 19, 235–249. doi: 10.1038/nrn.2018.19, 29515192

[ref70] Makrygianni E. A. Chrousos G. P. (2023). Extracellular vesicles and the stress system. Neuroendocrinology 113, 120–167. doi: 10.1159/000527182, 36137504

[ref71] Mazumder A. G. Julé A. M. Cullen P. F. Sun D. (2022). Astrocyte heterogeneity within white matter tracts and a unique subpopulation of optic nerve head astrocytes. iScience. 25:105568. doi: 10.1016/j.isci.2022.105568, 36465110 PMC9708923

[ref72] Mederos S. González-Arias C. Perea G. (2018). Astrocyte-neuron networks: a multilane highway of signaling for homeostatic brain function. Front. Synaptic Neurosci. 10:45. doi: 10.3389/fnsyn.2018.00045, 30542276 PMC6277918

[ref73] Mederos S. Sánchez-Puelles C. Esparza J. Valero M. Ponomarenko A. Perea G. (2021). GABAergic signaling to astrocytes in the prefrontal cortex sustains goal-directed behaviors. Nat. Neurosci. 24, 82–92. doi: 10.1038/s41593-020-00752-x, 33288910

[ref74] Mi Y. Qi G. Vitali F. Shang Y. Raikes A. C. Wang T. . (2023). Loss of fatty acid degradation by astrocytic mitochondria triggers neuroinflammation and neurodegeneration. Nat. Metab. 5, 445–465. doi: 10.1038/s42255-023-00756-4, 36959514 PMC10202034

[ref75] Moore C. S. Abdullah S. L. Brown A. Arulpragasam A. Crocker S. J. (2011). How factors secreted from astrocytes impact myelin repair. J. Neurosci. Res. 89, 13–21. doi: 10.1002/jnr.22482, 20857501

[ref76] Nakajima H. Fujita S. Kakae M. Nagayasu K. Oh-Hora M. Shirakawa H. . (2022). Orai2 channel regulates prostaglandin E2 production in TNFα/IL1α-stimulated astrocytes. Glia 70, 1666–1680. doi: 10.1002/glia.24188, 35506586

[ref77] Nash B. Thomson C. E. Linington C. Arthur A. T. McClure J. D. McBride M. W. . (2011). Functional duality of astrocytes in myelination. J. Neurosci. 31, 13028–13038. doi: 10.1523/JNEUROSCI.1449-11.2011, 21917786 PMC6623277

[ref78] Nave K. A. Werner H. B. (2014). Myelination of the nervous system: mechanisms and functions. Annu. Rev. Cell Dev. Biol. 30, 503–533. doi: 10.1146/annurev-cellbio-100913-013101, 25288117

[ref79] Nedergaard M. (2013). Neuroscience. Garbage truck of the brain. Science 340, 1529–1530. doi: 10.1126/science.1240514, 23812703 PMC3749839

[ref80] Ngoc K. H. Jeon Y. Ko J. Um J. W. (2025). Multifarious astrocyte-neuron dialog in shaping neural circuit architecture. Trends Cell Biol. 35, 74–87. doi: 10.1016/j.tcb.2024.05.002, 38853082

[ref81] Oberheim N. A. He W. Lin J. H. Wang F. Xu Q. Wyatt J. D. . (2009). Uniquely hominid features of adult human astrocytes. J. Neurosci. 29, 3276–3287. doi: 10.1523/JNEUROSCI.4707-08.2009, 19279265 PMC2819812

[ref82] Oksanen M. Hyötyläinen I. Trontti K. Wojciechowski S. Koskuvi M. Viitanen M. . (2020). NF-E2-related factor 2 activation boosts antioxidant defenses and ameliorates inflammatory and amyloid properties in human Presenilin-1 mutated Alzheimer’s disease astrocytes. Glia 68, 589–599. doi: 10.1002/glia.23741, 31670864 PMC7003860

[ref83] Panatier A. Vallée J. Haber M. Murai K. K. Lacaille J. C. Robitaille R. (2011). Astrocytes are endogenous regulators of basal transmission at central synapses. Cell 146, 785–798. doi: 10.1016/j.cell.2011.07.022, 21855979

[ref84] Panattoni G. Amoriello R. Memo C. Thalhammer A. Ballerini C. Ballerini L. (2021). Diverse inflammatory threats modulate astrocytes Ca2+ signaling via connexin43 hemichannels in organotypic spinal slices. Mol. Brain 14:159. doi: 10.1186/s13041-021-00868-6, 34696792 PMC8547100

[ref85] Patani R. Hardingham G. E. Liddelow S. A. (2023). Functional roles of reactive astrocytes in neuroinflammation and neurodegeneration. Nat. Rev. Neurol. 19, 395–409. doi: 10.1038/s41582-023-00822-1, 37308616

[ref86] Patel M. R. Weaver A. M. (2021). Astrocyte-derived small extracellular vesicles promote synapse formation via fibulin-2-mediated TGF-β signaling. Cell Rep. 34:108829. doi: 10.1016/j.celrep.2021.108829, 33691102 PMC8002899

[ref87] Perea G. Sur M. Araque A. (2014). Neuron-glia networks: integral gear of brain function. Front. Cell. Neurosci. 8:378. doi: 10.3389/fncel.2014.00378, 25414643 PMC4222327

[ref88] Petroccione M. A. Todd G. C. Wisnoski J. J. De Guzman R. M. Migliore R. Khmaladze A. . (2020). Circadian modulation of neurons and astrocytes controls synaptic plasticity in hippocampal area CA1. Cell Rep. 33:108255. doi: 10.1016/j.celrep.2020.108255, 33053337 PMC7700820

[ref89] Plog B. A. Nedergaard M. (2018). The glymphatic system in central nervous system health and disease: past, present, and future. Annu. Rev. Pathol. 13, 379–394. doi: 10.1146/annurev-pathol-051217-111018, 29195051 PMC5803388

[ref90] Prabhakar P. Pielot R. Landgraf P. Wissing J. Bayrhammer A. van Ham M. . (2023). Monitoring regional astrocyte diversity by cell type-specific proteomic labeling in vivo. Glia 71, 682–703. doi: 10.1002/glia.24304, 36401581

[ref91] Prada I. Marchaland J. Podini P. Magrassi L. D'Alessandro R. Bezzi P. . (2011). REST/NRSF governs the expression of dense-core vesicle gliosecretion in astrocytes. J. Cell Biol. 193, 537–549. doi: 10.1083/jcb.201010126, 21536750 PMC3087003

[ref92] Qian X. Song H. Ming G. L. (2019). Brain organoids: advances, applications and challenges. Development 146:dev166074. doi: 10.1242/dev.166074, 30992274 PMC6503989

[ref93] Rasmussen M. K. Mestre H. Nedergaard M. (2018). The glymphatic pathway in neurological disorders. Lancet Neurol. 17, 1016–1024. doi: 10.1016/S1474-4422(18)30318-1, 30353860 PMC6261373

[ref94] Recasens M. Shrivastava K. Almolda B. González B. Castellano B. (2019). Astrocyte-targeted IL-10 production decreases proliferation and induces a downregulation of activated microglia/macrophages after PPT. Glia 67, 741–758. doi: 10.1002/glia.23573, 30548340

[ref95] Rong Y. Ji C. Wang Z. Ge X. Wang J. Ye W. . (2021). Small extracellular vesicles encapsulating CCL2 from activated astrocytes induce microglial activation and neuronal apoptosis after traumatic spinal cord injury. J. Neuroinflammation 18:196. doi: 10.1186/s12974-021-02268-y, 34511129 PMC8436564

[ref96] Rothhammer V. Borucki D. M. Tjon E. C. Takenaka M. C. Ardura-Fabregat A. Staszewski O. . (2018). Microglial control of astrocytes in response to microbial metabolites. Nature 557, 724–728. doi: 10.1038/s41586-018-0119-x, 29769726 PMC6422159

[ref97] Rupprecht P. Duss S. N. Becker D. Lewis C. M. Bohacek J. Helmchen F. (2024). Centripetal integration of past events in hippocampal astrocytes regulated by locus coeruleus. Nat. Neurosci. 27, 927–939. doi: 10.1038/s41593-024-01612-8, 38570661 PMC11089000

[ref98] Sanmarco L. M. Polonio C. M. Wheeler M. A. Quintana F. J. (2021). Functional immune cell-astrocyte interactions. J. Exp. Med. 218:e20202715. doi: 10.1084/jem.20202715, 34292315 PMC8302447

[ref99] Schober A. L. Wicki-Stordeur L. E. Murai K. K. Swayne L. A. (2022). Foundations and implications of astrocyte heterogeneity during brain development and disease. Trends Neurosci. 45, 692–703. doi: 10.1016/j.tins.2022.06.009, 35879116

[ref100] Schouten P. Sinnema M. Stumpel C. T. R. M. Curfs L. M. G. Höybye C. Meijer O. C. . (2025). Selective changes in vasopressin neurons and astrocytes in the suprachiasmatic nucleus of Prader-Willi syndrome subjects. J. Neuroendocrinol. 37:e70015. doi: 10.1111/jne.70015, 40055943 PMC12045672

[ref101] Shaw B. C. Kaiser K. E. Smith B. C. Timken J. P. Louveau A. Williams J. L. (2026). Astrocytes upregulate interleukin-15 in response to neuroinflammation during Alzheimer’s disease. J. Alzheimer's Dis 109, 224–234. doi: 10.1177/13872877251393275, 41191004 PMC12750416

[ref102] Shen X. Y. Gao Z. K. Han Y. Yuan M. Guo Y. S. Bi X. (2021). Activation and role of astrocytes in ischemic stroke. Front. Cell. Neurosci. 15:755955. doi: 10.3389/fncel.2021.755955, 34867201 PMC8635513

[ref103] Sloan S. A. Darmanis S. Huber N. Khan T. A. Birey F. Caneda C. . (2017). Human astrocyte maturation captured in 3D cerebral cortical spheroids derived from pluripotent stem cells. Neuron 95, 779–790.e6. doi: 10.1016/j.neuron.2017.07.035, 28817799 PMC5890820

[ref104] Stephenson J. Nutma E. van der Valk P. Amor S. (2018). Inflammation in CNS neurodegenerative diseases. Immunology 154, 204–219. doi: 10.1111/imm.12922, 29513402 PMC5980185

[ref105] Tewari B. P. Woo A. M. Prim C. E. Chaunsali L. Patel D. C. Kimbrough I. F. . (2024). Astrocytes require perineuronal nets to maintain synaptic homeostasis in mice. Nat. Neurosci. 27, 1475–1488. doi: 10.1038/s41593-024-01714-3, 39020018 PMC11303255

[ref106] Torres-Ceja B. Olsen M. L. (2022). A closer look at astrocyte morphology: development, heterogeneity, and plasticity at astrocyte leaflets. Curr. Opin. Neurobiol. 74:102550. doi: 10.1016/j.conb.2022.102550, 35544965 PMC9376008

[ref107] Tran L. T. Yang D. J. Ha T. T. A. Mai T. D. Kim S. K. DePinho R. A. . (2023). Astrocytic FoxO1 in the hypothalamus regulates metabolic homeostasis by coordinating neuropeptide Y neuron activity. Glia 71, 2735–2752. doi: 10.1002/glia.24448, 37655904

[ref108] Wang L. Guo Q. Acharya S. Zheng X. Huynh V. Yimit A. . (2024). Primary cilia signaling in astrocytes mediates development and regional-specific functional specification. Nat. Neurosci. 27, 1708–1720. doi: 10.1038/s41593-024-01726-z, 39103557

[ref109] Wheeler M. A. Clark I. C. Tjon E. C. Li Z. Zandee S. E. J. Watson B. R. . (2020). MAFG-driven astrocytes promote CNS inflammation. Nature 578, 593–599. doi: 10.1038/s41586-020-1999-0, 32051591 PMC8049843

[ref110] Xie C. Lin Y. Qi C. Wang W. Yuan Y. Wang Z. . (2025). Neuro-endocrine-immune regulation of metabolic homeostasis. Cytokine Growth Factor Rev. 85, 165–178. doi: 10.1016/j.cytogfr.2025.08.001, 40816942

[ref111] Xing C. Li W. Deng W. Ning M. Lo E. H. (2018). A potential gliovascular mechanism for microglial activation: differential phenotypic switching of microglia by endothelium versus astrocytes. J. Neuroinflammation 15:143. doi: 10.1186/s12974-018-1189-2, 29764475 PMC5952884

[ref112] Yang L. Qi Y. Yang Y. (2015). Astrocytes control food intake by inhibiting AGRP neuron activity via adenosine A1 receptors. Cell Rep. 11, 798–807. doi: 10.1016/j.celrep.2015.04.002, 25921535

[ref113] Yin H. Zhang X. Liu Y. Zhang Y. Ci H. Li K. . (2026). The CCL11-CCR3 axis regulates the aggravation of neuroinflammation in astrocytic necroptosis after cerebral ischemia. FASEB J. 40:e71373. doi: 10.1096/fj.202502723RR, 41447092 PMC12739800

[ref114] You Y. Borgmann K. Edara V. V. Stacy S. Ghorpade A. Ikezu T. (2020). Activated human astrocyte-derived extracellular vesicles modulate neuronal uptake, differentiation and firing. J. Extracell. Vesicles 9:1706801. doi: 10.1080/20013078.2019.1706801, 32002171 PMC6968484

[ref115] Zhang L. Jia Z. Cai S. Wu Q. Hu X. Bai T. . (2025). Modulating mTOR-dependent astrocyte substate transitions to alleviate neurodegeneration. Nature Aging 5, 468–485. doi: 10.1038/s43587-024-00792-z, 39779911

[ref116] Zhang Y. M. Qi Y. B. Gao Y. N. Chen W. G. Zhou T. Zang Y. . (2023). Astrocyte metabolism and signaling pathways in the CNS. Front. Neurosci. 17:1217451. doi: 10.3389/fnins.2023.1217451, 37732313 PMC10507181

[ref117] Zhang Y. Sloan S. A. Caneda C. Plaza C. A. Blumenthal P. D. Vogel H. . (2016). Purification and characterization of progenitor and mature human astrocytes reveals transcriptional and functional differences with mouse. Neuron 89, 37–53. doi: 10.1016/j.neuron.2015.11.013, 26687838 PMC4707064

[ref118] Zhou J. Geng Y. Su T. Wang Q. Ren Y. Zhao J. . (2022). NMDA receptor-dependent prostaglandin-endoperoxide synthase 2 induction in neurons promotes glial proliferation during brain development and injury. Cell Rep. 38:110557. doi: 10.1016/j.celrep.2022.110557, 35354047

[ref119] Ziff O. J. Clarke B. E. Taha D. M. Crerar H. Luscombe N. M. Patani R. (2022). Meta-analysis of human and mouse ALS astrocytes reveals multi-omic signatures of inflammatory reactive states. Genome Res. 32, 71–84. doi: 10.1101/gr.275939.121, 34963663 PMC8744676

